# National biodiversity data infrastructures: ten essential functions for science, policy, and practice

**DOI:** 10.1093/biosci/biae109

**Published:** 2024-10-30

**Authors:** Anton Güntsch, Jörg Overmann, Barbara Ebert, Aletta Bonn, Yvan Le Bras, Thore Engel, Knut Anders Hovstad, Dora Ann Lange Canhos, Peggy Newman, Elaine van Ommen Kloeke, Sophia Ratcliffe, Marianne le Roux, Vincent S Smith, Dagmar Triebel, David Fichtmueller, Katja Luther

**Affiliations:** Center for Biodiversity Informatics and Collection Data Integration at Botanic Garden and Botanical Museum Berlin, Freie Universität Berlin Berlin, Germany; Leibniz Institute DSMZ-German Collection of Microorganisms and Cell Cultures; Microbiology at the Technical University of Braunschweig; German Federation for Biological Data and the NFDI4Biodiversity consortium; Biodiversity and People at the Helmholtz-Center for Environmental Research - UFZ, Friedrich Schiller University Jena; German Center for integrative Biodiversity Research (iDiv); French Biodiversity Data Hub (PNDB) e-infrastructure, PatriNat support and research unit, French Museum of Natural History, Concarneau marine station, France; Biodiversity and People at the Friedrich Schiller University Jena, the German Center for integrative Biodiversity Research (iDiv), Helmholtz-Center for Environmental Research - UFZ; Norwegian Biodiversity Information Centre, Centre for Biodiversity Dynamics, Norwegian University of Science and Technology, Norway; CRIA - Centro de Referência em Informação Ambiental, Campinas, São Paulo, Brazil; Atlas of Living Australia, Commonwealth Scientific and Industrial Research Organization (CSIRO) Melbourne; ARISE, Naturalis Biodiversity Center Leiden, the Netherlands; NBN Trust, NBN Atlas; e-Flora, South African National Biodiversity Institute, Pretoria; University of Johannesburg, Johannesburg, South Africa; Digitial, Data, and Informatics, Natural History Museum, London, England, United Kingdom; State Collection for Botany, SNSB IT Center Core Facility at the Bavarian Natural History Collections, Germany; Center for Biodiversity Informatics and Collection Data Integration at Botanic Garden and Botanical Museum Berlin, Freie Universität Berlin Berlin, Germany; Center for Biodiversity Informatics and Collection Data Integration at Botanic Garden and Botanical Museum Berlin, Freie Universität Berlin Berlin, Germany

**Keywords:** biodiversity data, CBD, data mobilization, open data, biodiversity informatics

## Abstract

Today, at the international level, powerful data portals are available to biodiversity researchers and policymakers, offering increasingly robust computing and network capacities and capable data services for internationally agreed-on standards. These accelerate individual and complex workflows to map data-driven research processes or even to make them possible for the first time. At the national level, however, and alongside these international developments, national infrastructures are needed to take on tasks that cannot be easily funded or addressed internationally. To avoid gaps, as well as redundancies in the research landscape, national tasks and responsibilities must be clearly defined to align efforts with core priorities. In the present article, we outline 10 essential functions of national biodiversity data infrastructures. They serve as key providers, facilitators, mediators, and platforms for effective biodiversity data management, integration, and analysis that require national efforts to foster biodiversity science, policy, and practice.

The Kunming–Montreal Global Biodiversity Framework (GBF) sets ambitious goals including global biodiversity monitoring (CBD 2022). However, the current means to assess successful biodiversity policy implementation or to inform on effective strategies are very limited because of a lack of national biodiversity data infrastructures (NBDIs) in many countries (Xu et al. 2021). As a result, fragmented landscapes for biodiversity data, a lack of data harmonization, and ineffective data access flows impede policy uptake of biodiversity data (Kühl et al. 2020, Moersberger et al. 2024). Already in 1992, at the United Nations Conference on Environment and Development in Rio de Janeiro, Brazil, the resulting Convention on Biological Diversity (CBD) called for biodiversity data infrastructures in article 17:

“The Contracting Parties shall facilitate the exchange of information, from all publicly available sources, relevant to the conservation and sustainable use of biological diversity, taking into account the special needs of developing countries.”

The GBF emphasizes the need for improved data collection, monitoring, and information sharing at the national level to support decision-making. The framework can guide further development and harmonization of national data collection and reporting efforts using a standard set of essential biodiversity variables that directly informs indicators for monitoring change in biodiversity and ecosystem function (Perino et al. 2022, Gonzalez et al. 2023). The approach of the Kunming–Montreal GBF can therefore initiate further stimulation and development of NBDIs.

The establishment of the CBD triggered a number of initiatives to develop NBDIs in different parts of the world, and an informal collaborative network was established including countries such as Brazil, Australia, Ecuador, Costa Rica, and Finland (Biodiversity Information Network 21).

For many decades, the management and storage of biodiversity data have been an important responsibility of the environmental sciences. Data categories processed are highly diverse and include, among others, information on preserved collections (natural history museums, herbaria, DNA banks, etc.), on living collections (botanical and zoological gardens, as well as microbial or plant culture collections), data from floristic and faunistic monitoring, citizen science, literature, multimedia, and traits, as well as sequence-related information (Berendsohn et al. 2011).

With the turn of the millennium and the increased introduction of web services and service-oriented architectures, data management in biodiversity research has moved away from one-dimensional, locally operating platforms to specialized services that are developed in a division of labor and made available worldwide. The rapidly growing network and computing capacities and the ongoing standardization of data formats and access protocols, driven by organizations such as Biodiversity Information Standards (TDWG), has yielded a range of powerful and robust data services at the international level. These range from data platforms for recording observations to aggregator services that provide standardized access to distributed biodiversity data, such as observations, sequences, specimens, and literature to services for accessing ontologies and taxonomic backbone systems. Several tasks, however, are difficult to carry out at the international level and require national efforts.

As a result of the 2nd Global Biodiversity Informatics Conference in 2018, 103 delegates from a wide range of global, national, and regional organizations and initiatives postulated a global alliance for biodiversity knowledge. They formulated 23 common goals that play a crucial role in supporting the Aichi Biodiversity Targets included in the CBD Strategic Plan for Biodiversity and whose implementation should be promoted within the framework of a global alliance (Hobern et al. 2019). These targets fall into the following categories: support for science and evidence-based planning (e.g., by providing a platform for continuous growth in understanding of biodiversity by preserving, building on and improving existing knowledge), support for open data and open science (e.g., through free and open sharing of data and adoption of FAIR data principles), support for highly connected biodiversity data (e.g., by enabling the combination, querying and analysis of different classes of biodiversity information as an interconnected whole), and support for international collaboration (e.g., through the development of flexible, collaborative approaches to designing, building and sustaining all components of this distributed knowledge infrastructure). The particular importance of national and regional initiatives is emphasized—for example, in the generation, analysis, and application of data and in the application of new infrastructure, tools, services, and practices to address national priorities.

To bridge the gaps between local institutional or project-related data management and internationally available infrastructures and to pursue specific goals that can be most effectively addressed within countries, data infrastructures were developed. Examples of national data infrastructures include the Atlas of Living Australia (ALA), NFDI4Biodiversity in Germany, the National Biodiversity Network Atlas in the United Kingdom, ARISE (the Authoritative and Rapid Identification System for Essential Biodiversity Information) in the Netherlands, the Swedish Biodiversity Data Infrastructure (SBDI), the Biodiversity Advisor in South Africa, the Integrated Digitized Biocollections (iDigBio) in the United States, *species*Link in Brazil, CONABIO (the Comisión Nacional para el Conocimiento y Uso de la Biodiversidad) in Mexico and the Norwegian Biodiversity Information Centre. These national initiatives feed data to the Global Biodiversity Information Facility (GBIF) and offer new insights as to data management and outputs. During a symposium at the 2022 TDWG Biodiversity Information Standards Conference (Luther and Güntsch 2022), eight national data infrastructures were presented and their overlapping but also very different focuses were discussed and synthesized. The initially Eurocentric composition of the symposium contributions was widened in the course of preparing this manuscript to include participants from Brazil and South Africa in order to provide a more global perspective. The participating infrastructures have a strong focus on the mobilization and digitization of observational and specimen primary data and the establishment of sustainable publication and analysis pipelines. Their added value for emerging technologies such as AI, environmental DNA, or data mining in recent and historic text documents was not the focus of the present assessment, but these are nevertheless rooted in the availability and reliability of primary data and the services, which infrastructures such as NBDI provide.

In the present article, we identify 10 key functions of national infrastructures for biodiversity data to provide guidance for their planning and development. By defining the 10 key NBDI functions, we also align and contrast them with the tasks of international data infrastructures to provide maximum synergy and joint effectiveness in biodiversity research and a foundation for policy.

## International biodiversity data infrastructures

Over the past decades, international initiatives have built numerous infrastructures to enable and support increasingly data-driven biodiversity research, i.e., analyses of large amounts of data rather than direct observations in the field or in laboratory experiments. The diverse services range from access to data aggregations, to computing services software tools that are made freely available, often via application programming interfaces (APIs). The publication of data according to FAIR principles (for *findable, accessible, interoperable*, and *reproducible*) is becoming increasingly important, because this is the only way to fully exploit the potential of existing research data for subsequent use and integration (Wilkinson et al. 2016). The use of persistent and actionable identifiers for essential object classes is indispensable for stable referencing and integration of biodiversity data from different sources but is not fully established yet. Promising approaches exist—for example, for scientific names (Borsch et al. 2020), specimens (Güntsch et al. 2017, Lannom et al. 2022), and people (Groom et al. 2020). The adoption of the FAIR principles combined with the use of persistent identifiers, facilitates data integration and strengthens the construction of knowledge graphs that interconnect data originating from disparate sources and domains (Page 2019, Penev et al. 2022). The following examples illustrate the landscape of international biodiversity data infrastructures and services. Comprehensive overviews and assessments have been provided by Bingham and colleagues (2017) and Smith and colleagues (2022).

### The Global Biodiversity Information Facility

GBIF is an international network and data infrastructure funded by the member states to provide open access to all types of biodiversity data and support research, policy development, and decision-making (Edwards 2004). GBIF has also promoted and extended standards for interoperability, agreed on data pipelines, established the GBIF taxonomic backbone, and secured several software tools and training programs that are now used extensively by national biodiversity infrastructures—for example, for exchange of biodiversity data between institutions (Robertson et al. 2014). GBIF draws on the technical infrastructure of a network of established organizations as data publishers. Most of the GBIF participant nodes are national nodes, some are associate international and intergovernmental participant organizations. The GBIF nodes communicate biodiversity data strategies and concepts of the individual countries and organizations to the GBIF secretariat and coordinate GBIF-related in-country activities. In certain countries such as Australia, nodes are legal entities hosting a platform and primary portal for biodiversity data and therefore build their NBDI with specific tools and long-term data preservation perspectives. GBIF training programs are set up by the GBIF secretariat as well as GBIF members.

### Biodiversity Heritage Library

The Biodiversity Heritage Library (BHL) is the world's largest open access digital library for biodiversity literature. As of 2022, the BHL portal gives free access to 337,827 articles, 289,274 volumes and a total of 60,584,778 pages of biodiversity literature from the fifteenth to the twenty-first centuries (BHL 2022). In addition to the search capabilities in the portal, the BHL offers a number of powerful tools to integrate content into service-based workflows and platforms. These include content download services, scientific name tools, and DOIs, among others. Since its launch in 2007, the BHL has become a unique and indispensable infrastructure component of international biodiversity informatics, integrated into countless software systems, thanks to its comprehensive range of freely accessible literature and seamlessly integrable web services.

### International Nucleotide Sequence Database Collaboration and the European life sciences infrastructure ELIXIR

The International Nucleotide Sequence Database Collaboration (INSDC), with its vast collection of molecular data, is an important corpus of reference data for research and studies on genetic diversity and is funded by both national and European public-funding organizations on behalf of the global research community. Three databases in Japan (the DNA Data Bank of Japan), the United States (GenBank), and Europe (the European Nucleotide Archive, ENA) hold synchronized data collections, which are submitted to and curated decentrally by individual researchers all over the world, on the basis of INSDC’s submission standards. The ENA is part of the European Research Infrastructure ELIXIR, which curates a portfolio of essential resources for life sciences (Drysdale et al. 2020). A biodiversity user community has formed around the ELIXIR platforms for tools, training and computation (Waterhouse et al. 2023). ELIXIR operates through a network of national nodes and has a central office, which is funded by member countries. The example of ELIXIR shows how national resources are consolidated and help sustain joint international initiatives.

### Data Observation Network for Earth

The Data Observation Network for Earth (DataONE; Michener at al.^ 2011^) is a community-driven program providing access to data across multiple member repositories, supporting enhanced search and discovery of Earth and environmental data. DataONE promotes best practices in data management and supports researchers, educators, and the public to better understand and conserve life on Earth and the environment that sustains it. Started in 2009, DataONE is now managed through the National Center for Ecological Analysis and Synthesis at University of California, Santa Barbara, in the United States. With 44 member repositories sharing data, infrastructure, and expertise, the DataONE network gives access to a total of more than 840,000 data sets representing 83 terabytes of data shared in raw formats (tabular, spatial vector, audio, netCDF, or others) with the possibility to also share source code for scientific software. Recently, an assessment score for all metadata based on FAIR principles was developed.

### iNaturalist

iNaturalist is an America-based nonprofit organization, online data network, and international citizen science platform. The international focus is supplied by a network of more than 20 member nodes who manage their own websites and a range of activities to provide a regional focus to the international platform and database. The members curate localized portals with relevant threatened species information, provide user support, and engage with local governments and authorities on behalf of the platform. The rapid growth in the iNaturalist data set has a recursive effect on the quality of data produced by the platform, because increased volumes of identified observations improve the accuracy of the computer vision model, which, in turn, supports improved identifications. Mobile phone applications are available and are used by citizen science groups worldwide.

iNaturalist has taken a crowdsourcing approach to developing its taxonomic backbone as a means of tackling the challenges of building and maintaining a global taxonomy. iNaturalist provides guidance for updating taxonomy, and the work is managed by the taxon curator community. iNaturalist encourages data sharing and FAIR principles through the use of Creative Commons licensing and shares openly available data with GBIF and the living atlases via their own APIs.

### Catalogue of Life

The Catalogue of Life is an international effort with the aim of providing a global list of all species (Hobern et al. 2021). The data foundation for the Catalogue of Life is formed from diverse curated species lists, which can originate from data contributions of individual experts and expert groups but also from large networks with complex editorial workflows, depending on the taxonomic group. As of February 2024, the Catalogue of Life includes 2.15 million accepted species, both living and extinct (https://www.catalogueoflife.org/2024/02/22/release).

The technical Catalogue of Life infrastructure is hosted by GBIF and includes, next to the data portal, a growing number of tools for data integration, data quality control, and mechanisms for referencing taxa via stable identifiers. An outstanding feature of the infrastructure is the Catalogue of Life Checklistbank, which allows thematic and regional species lists to be published and reused in a standardized format. The Catalogue of Life provides the taxonomic backbone for numerous national and international data portals and is therefore an important component for the integration and analysis of biodiversity-related research data.

### Scope and limits of international data infrastructures

International data infrastructures have been set up and operated collaboratively over a significant period of time. They invariably act as data distributors and data curators (table 1), integrating data and information from a wide range of providers, from individuals to public research institutions all over the world. Although there are single responsible institutions for each of them, the operation is usually a collaborative effort of publicly funded partners and, in some cases, of pooled (national) funding. Despite their long history, reliable resourcing for these international data infrastructures is challenging, with many infrastructures relying on in-kind contributions from supportive national institutions. The Global Biodata Coalition is an effort to pave the way for persistent international alliances to secure core biodata resources in the long term.

**Table 1.** Scope of selected international biodiversity infrastructure.

Infrastructure	Focus data type	Category	Funding and operation	Responsible institution
GBIF	Species occurrence data	Data curator, data distributor	Public, nonprofit, US organization	GBIF secretariat (intergovernmental institution)
BHL	Literature	Data distributor	Public, nonprofit, US organization	BHL consortium @Smithsonian Libraries and archives
INSDC	Molecular sequence data	Data curator, data distributor	Public, nonprofit, international contracts	INSDC consortium
DataONE	Earth and environmental data	Data distributor	Public, nonprofit, US authority	National Center for Ecological Analysis and Synthesis at UC Barbara
iNaturalist	Species observations data	Data creator, data distributor	Private nonprofit, US organization	California Academy of Sciences
Catalogue of Life	Taxonomic data	Data curator, data distributor	Public nonprofit, intergovernmental organization	Stichting Catalogue of Life

## Evolution of national biodiversity data infrastructures

The use of digital databases to store and systemize records of species occurrences and museum specimens began in the late 1970s (Bisby 2000). An increasing awareness of the loss of species and habitats created a need to compile and share data on species diversity at a larger scale, and in the late 1980s, several initiatives started to build larger infrastructures for biodiversity data (Nelson and Ellis 2018, Peterson et al. 2023). In Costa Rica, the Instituto Nacional de Biodiversidad (INBio) was established in 1989 with an ambitious goal to conduct a complete inventory of biological diversity in the country (Tangley 1990, Sandlund 1991). INBio pioneered the development of computer systems and databases for biodiversity data, and its establishment is one of the first milestones in the evolution of the NBDIs of today (Tangley 1990). Three years later, the UN Earth Summit conference in Rio de Janeiro initiated the CBD, which explicitly pointed to the need to develop data infrastructures.

In the runup to the conference, Mexico established CONABIO to coordinate and collate biodiversity data and assessments in the country (Sarukhán and Jiménez 2016, Soberon 2022). In Brazil, *species*Link was established as a direct result of the UN conference (Canhos et al. 2022). The development of these national data infrastructures was guided by two main principles: The data should be primary biodiversity data with analysis and interpretation of the data being the responsibility of the user and data should be openly accessible for everyone. These principles became the foundation for the development of other, subsequent NBDIs.

To further facilitate access to biodiversity data and international cooperation, GBIF was founded in 2001 (Nelson and Ellis 2018). Its establishment followed a recommendation by the Organisation for Economic Co-operation and Development's Megascience Forum's working group on biological informatics to create “an international mechanism… to make biodiversity data and information accessible worldwide” (OECD 1999). In the same year, the Australian Virtual Herbarium was created, and this infrastructure was one of the predecessors for the ALA, which was established in 2007 (Belbin et al. 2021). The ALA builds extensively on international standards such as Darwin Core and the GBIF infrastructure and has had a large influence, both conceptually and technically, on the development of other NBDI's and initiated the development of the international living atlas community (Brenton et al. 2018, Belbin et al. 2021).

*species*Link was launched online in 2002 to integrate data from Brazilian biological collections. This network evolved and developed a number of tools such as data cleaning, indicators, and systems to identify data gaps and produce ecological niche models. Besides offering data to GBIF and later, iDigBio, among others, *species*Link also integrates data from these infrastructures. Important partnerships in Brazil were established with the botanical community, producing the Brazilian virtual herbarium and a network of bee collections. More recently, important partnerships were established with MapBiomas (land use and coverage data) and the Google Cloud Platform. All systems are integrated through *species*Link’s search interface (https://specieslink.net/search/).

More recent examples of national initiatives include NFDI4Biodiversity in Germany, the ARISE initiative in the Netherlands and the SBDI. In Germany, NFDI4Biodiversity (Glöckner et al. 2020) is part of the German National Research Data Infrastructure (NFDI). The NFDI serves to coordinate the investments and individual digitization and data strategies of the federal government and the governments of the 16 länder, in order to ensure FAIR access to essential data services for scientists across all German universities and research organizations. Its initial 5-year work program builds on previous work of the German Federation for Biological Data (GFBio), the German GBIF nodes, as well as 26 use cases, one of which is a feasibility study for Living Atlas of Nature in Germany (Bonn et al. 2016). Taking a user-centered approach, NFDI4Biodiversity establishes effective workflows for data integration and harmonization, analysis, and visualization to further data providers and users. A GBIF hosted portal currently serves to showcase national biodiversity (https://land.gbif.de/).

In the Netherlands, ARISE kickstarted in 2020, funded for 10 years by the Dutch Research Council, and is built by a partnership between the Naturalis Biodiversity Center, the Westerijk Fungal Institute, the University of Amsterdam, and the University of Twente, which each bring their own expertise on biodiversity and data. The main goal of ARISE is to establish an end-to-end infrastructure to enable the recognition of any species in any location, using digital sensors, DNA or eDNA, and AI. A key focus is also delivering user friendly end-user services (e-services) for all users, ranging from citizen to data scientist. Learning from existing global and national initiatives, the ARISE program spearheads the national innovation and further development of all dimensions of species recognition in order to drive biodiversity monitoring at scale for the research community and beyond.

The SBDI aims to provide researchers with unified and open access to biodiversity data and to a wide range of tools for querying, visualizing, and analyzing these data (SBDI 2021). The SBDI is funded by the Swedish Research Council and 11 organizations forming the SBDI consortium. Employing the living atlases software, the SBDI provides tools for querying, visualizing and analyzing biodiversity data.

In the United States, iDigBio, funded by the National Science Foundation, coordinates the digitization of biological specimens (data and images) on a national level. iDigBio currently serves about 142 million specimen-based records and 57 million media records from about 1850 data sets. iDigBio, together with the European DiSSCo network (Distributed System of Scientific Collections), is involved in an initiative that aims to create a new interconnected network of digital extended specimens on the Internet (Hardisty et al. 2022). Digital extended specimens are digital images of the physical specimens in collections and provide the basis for the digital interoperable linking of collection data with other data domains.

The South African National Biodiversity Institute (SANBI) is a government funded organization with the assignment under the South African National Environmental Management: Biodiversity Act 10 (South African government 2004) to study, coordinate, promote, and disseminate information on the South African biodiversity. In response to this, SANBI established a centralized platform, the Biodiversity Advisor, to disseminate all data held by SANBI and its partner institutions across the country through an open-access, interactive, and user-friendly interface. The data shared include occurrence data, checklists, floristic and faunistic data, DNA data, threat statuses, literature, images, and derived geospatial data. The Biodiversity Advisor aims to promote quality improvement, standardization, and attribution to enable knowledge sharing within the biodiversity data community and beyond.

These are examples of initiatives created to organize and serve biodiversity data for research, education, and policy mostly on the basis of specimen data from biological collections. There are a number of existing initiatives that also include observation data such as eBird and iNaturalist, among many others.

## Roles of national biodiversity data infrastructures

**Figure 1.**
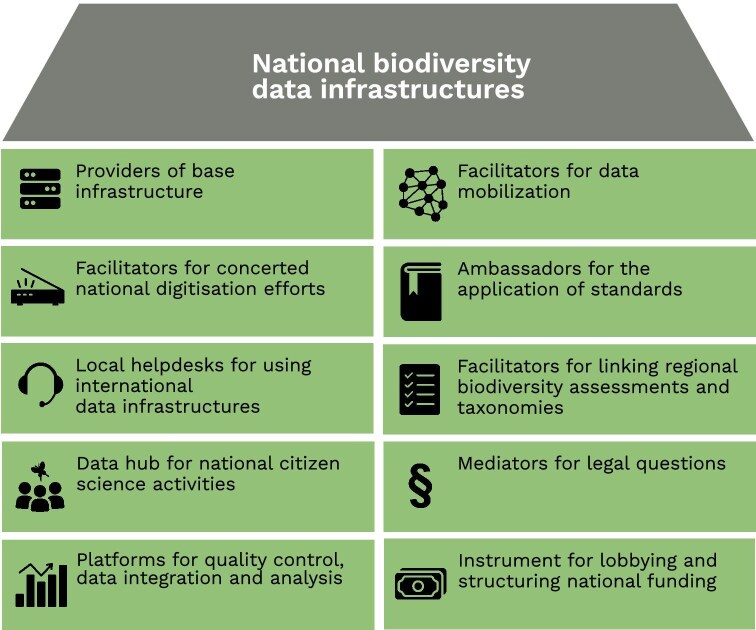
10 essential functions of national biodiversity data infrastructures.

Despite a rapidly developing landscape of international biodiversity data infrastructures, multiple responsibilities remain that can be better and more effectively—and in some cases, even exclusively—fulfilled at the national level. In the following, we define 10 essential functions of national data infrastructures (figure 1). Complementary to the functions of international data infrastructures, these outline the most important roles of NBDIs and are intended to provide guidance for the future development of national structures, to determine priorities, and to foster cooperation.

### NBDIs as providers of base infrastructure

International infrastructures are focused on the mobilization, aggregation, and publication of biodiversity data but do not provide the basic infrastructure needed for data and metadata preservation and high-performance computing. Funding of the required computing capacities is mainly in national hands.

*Long-term data preservation and archiving* refers to data preservation on dedicated storage systems, which guarantees long-term availability. This includes bitstream archiving, as well as functional archiving, which is not fully covered by international infrastructure services. Data storage and preservation include the assignment of persistent identifiers, with access often depending on institutional commitments of data providers or data publishers at a national and regional level. In particular, this is an issue for original primary field-based biodiversity data that are valuable references for subsequent environmental studies.

The data sets used by biodiversity scientists are constantly growing, with more and more recorders providing huge data sets (including nucleotidic sequencing or mass spectrometry platforms for Omics data, devices to capture sounds, pictures, or videos). In addition, researchers combine biodiversity data with auxiliary big data sets—for example, produced by satellite remote-sensing technologies. In parallel, artificial intelligence (AI) algorithms are now used as regular analytical tools requiring high-performance computing services. NBDIs have an important role as a translator between users and national high-performance computing facilities not only on data science-oriented processes but also in administrative matters, as well as guiding users to appropriate computing resources and to support national domain-specific data trustees and archival institutions (Markus et al. 2024).

### NBDIs as facilitators for data mobilization

Biodiversity research increasingly relies on the availability and analysis of big data to generate novel knowledge. So far, biodiversity data are still poorly accessible, often highly fragmented and dispersed across the literature, as well as in files and databases of public research institutes and government agencies (e.g., Kühl et al. 2020, Sweet et al. 2022), and, therefore, must be mobilized. Access to high-quality biodiversity data is not only key to gain novel insights in basic research but also highly relevant for conservation planning and national policy development and accountability, as well as the sustainable use of biodiversity and the development of national bioeconomies. Scientific discovery, research, and development can be significantly accelerated if sufficient molecular, biochemical, and phenotypic information on biological species becomes accessible and can be analyzed readily (e.g., Reimer et al. 2022).

In order to address these challenges, biodiversity data often need to be mobilized at the national level. In fact, international biodiversity data infrastructures such as GBIF typically represent networks of national nodes or institutions that are crucial for the mobilization of data. Importantly, efforts to halt and counteract the worldwide biodiversity loss require national biodiversity strategies and action plans (CBD 2023), which, in turn, depend on the availability of comprehensive, national biodiversity data. Notably, the lack of national or regional data integrators correlates with a strong geographical underrepresentation of biodiversity data in GBIF (Schulmann et al. 2021). As another example, national bioeconomies rely on the development of novel and sustainable applications for suitable organisms and therefore on biodiversity data that are often specific for a particular ecological and economical setting (e.g., the cocoa fermentation by microorganisms in a limited number of subtropical countries or the composition of the rhizobiome of specific crop plants). At the same time, national data sets need to be harmonized and redundancies avoided to conduct collaborative cross-border analyses (Novotný et al. 2022).

In order to enable large-scale biodiversity analysis, to ensure geographically representative monitoring, or to lay the foundation for bioeconomy (among other goals), all countries must have the chance to generate, store, and easily access their biodiversity data. Countries that encounter difficulties during the development of their own national NBDI might profit from the already established structures of a partner country. Although bilateral joint funding schemes would be needed to mobilize, digitize, and standardize data under the specific domestic conditions of the country in need and to adapt existing database structures to the priorities of another country, building on an existing and well-functioning infrastructure will be significantly more cost effective and much more rapid than starting alone and from scratch. The modified database structure in the partner country can then serve as a starting point for the subsequent buildup of an own national NBDI if needed. In a specific case, this model of support has successfully been realized by the Bac*Dive* database, in which large data sets of three other microbial cultures collections from other countries were integrated, now allowing joint analysis of all data (Reimer et al. 2022). As an additional benefit, Bac*Dive* contributes significantly to the visibility of these partners through direct links of data entries to the data providing institutions and by explicitly naming them.

To localize, filter, and structure biodiversity data, different scientific expertise is required and must be aligned, involving skills in biodiversity research, biodiversity monitoring, and collection and literature management, as well as data sciences. A critical mass of expertise can be reached by joining existing research infrastructures on a national level through the establishment of NBDIs. These can be capable of covering the demands of the different national sectors and at the same time provide a link to international data infrastructures. NBDIs are therefore established in a nationally coordinated, strategic approach in contrast to the numerous project-based initiatives that typically exist in parallel with little interlink or mutual benefit and often limited sustainability. In fact, consortia of research infrastructures dedicated to the gathering, maintenance, and provision of biodiversity information have recently led the national efforts to jointly and successfully establish NBDIs (Glöckner et al. 2020). NBDIs routinely mobilize and gather relevant biodiversity data and provide a wide array of services with relevance for national and international users alike. As a final aspect, the largest amount of infrastructure funding is usually supplied at the national level and often related to nature conservation aspects. This can be accessed most efficiently by the national research infrastructures in cooperation with regional environmental agencies, which also offer domain-specific expertise outside of academia and NBDIs thereby avoiding counterproductive competition and redundancy of smaller database projects.

In the context of data mobilization, NBDIs can play the role of data trustees. They mediate between local data producers and international infrastructures, validate data products, and ensure that data protection concerns and other legal requirements are adequately addressed, besides developing outputs of national interest.

### NBDIs as facilitators for concerted national digitization efforts

In some countries, the development of a national data portal for natural science collections has been closely aligned with efforts to develop a national digitization infrastructure. Examples are iDigBio in the United States and the Distributed System of Scientific Collections United Kingdom (DiSSCo UK; Smith et al. 2022). DiSSCo UK is a program of work to revolutionize how UK institutions manage, share, and use the United Kingdom’s natural science collections, creating a distributed network that provides a step change digitization and collections research infrastructure for the United Kingdom. Although the physical integration of UK natural science collections would be almost inconceivable, their digital integration through a national data portal is within reach. Building off of the UK Natural History Museum's digitization program and in partnership with more than 90 collection-holding institutions across the length and breadth of the United Kingdom, DiSSCo UK aims to unlock the scientific, economic and social benefits of the United Kingdom’s natural science collections, which are presently constrained by the limits of physical access. With just 8% of the United Kingdom’s 137 million specimens currently available digitally and many of these coming from the UK Natural History Museum's institutional data portal (https://data.nhm.ac.uk/), DiSSCo UK seeks to massively accelerate the digitization of these collections and the impact of these data through a nationally coordinated program.

To demonstrate this potential, the DiSSCo UK team constructed the first national data portal (https://dissco-uk.org/) for natural science collections in 2023, aggregating an initial 11 million records. This was developed using GBIF's hosted portal solution (https://www.gbif.org/hosted-portals/), which provides a customizable interface to filter data published to GBIF from selected UK data providers. A two-step process, first involves institutions registering themselves on GRSciColl (the Global Registry of Scientific Collections; https://scientific-collections.gbif.org/). GrSciColl is a community curated registry for natural science collections across the globe, and DiSSCo UK uses this tool to collate institutional level metadata on the scope and extent of their natural science collections. These data helped the UK team guide data mobilization efforts by determining how many active collections there are and the contents they hold.

The GBIF-hosted portal service has enabled the DiSSCo UK team to focus efforts on other critical activities such as generation of new data, adding more UK data publishers and data management.

### NBDIs as ambassadors for the application of standards

An essential success factor for international networking, accessibility, and interoperability of biodiversity data is standardization at various levels. For example, metadata standards such as Ecological Metadata Language or the Data Catalog Vocabulary and data standards such as Darwin Core (Wieczorek et al. 2012) and Access to Biological Collection Data (ABCD) allow the mapping of heterogeneous observation and collection data to uniform data definitions and then make them accessible via standardized service protocols (Berendsohn et al. 2011). Other standards that aid in the uniform processing of biodiversity data can relate, for example, to persistent identifier systems, ontologies, controlled vocabularies, and the selection of resources for semantic annotations (see, e.g., the application of the TWDG Plant Occurrence and Status Scheme (POSS) prestandard in a recent regional biodiversity project; Novotný et al. 2022).

In most cases, these standards are developed in international working groups in which experts prepare the standards and support the process of ratification and ongoing maintenance. For the correct application of standards and their consistent implementation at the local level, NBDIs play an important role by translating the often very technical specifications for use into practical research use cases, documenting aspects relevant to specific applications and providing a helpdesk for researchers. Conversely, national initiatives can bundle and document local needs and introduce them into international standardization processes.

### NBDIs as local helpdesks for using international data infrastructures

International data infrastructures such as DataONE or GBIF are sometimes seen as disconnected from local needs and users. National infrastructures can play an important role by promoting and supporting international initiatives that create bridges with local researchers so they can engage in these international infrastructures, such as through local working groups to express their distinct needs.

NBDIs offer a range of advantages for users who need help when interacting with an international data infrastructure. Although English is the default language for international scientific collaboration, the proficiency levels of users from non-English-speaking countries vary greatly and can be a hindrance for contribution, in particular if questions or problems arise that are outside of the default workflow for data contribution. In the present article, NBDIs can provide a service that would be very costly for international data initiatives to offer their users. Even if the website and support material is translated into multiple languages, usually only a limited number of languages are supported and translations can be outdated if the original material has been updated. In addition, helpdesk support staff are more likely to be familiar with national or regional practices or customs, as well as regulations and laws (see the section on NBDIs as mediators for legal questions), and can translate technical requirements into layman's terms suitable for relevant applications.

In order for NBDIs to efficiently function as helpdesks for international infrastructures, a broad and up-to-date knowledge set of the infrastructures, their functionalities, workflows, and support materials is required by the NBDIs. This will substantially enhance and alleviate the work of the international helpdesk of the infrastructure, which can then act as an overarching structure for both national and international success. Accordingly, GBIF has long encouraged the assistance for users of their infrastructure at a national level through their network of national nodes.

### NBDIs as facilitators for linking regional biodiversity assessments and taxonomies

There are interesting symbiotic relationships between global and regional taxonomies. Most biodiversity conservation initiatives are implemented in national government legislation, are conducted at a regional level, and most often cover threatened species and restricted-range species. The latter species are defined on the basis of local authoritative expertise and knowledge. Species determinations and classifications within a regional authority may be in conflict with a global taxonomic consensus. For this reason, NBDIs play a fundamental role in collating and translating localized taxonomic information, including indigenous knowledge and culture, that serves the purposes of active legislation and programs.

In turn, these localized taxonomies can inform parts of global taxonomies. For international taxonomies, they are invaluable for compiling traits and characteristics for invasive species for a region, assisting jurisdictions to implement management strategies for species not endemic to their region that pose biosecurity threats.

A key contribution of NBDIs is therefore the compilation and technical integration of national and even more regional taxonomic checklists, whose development and curation rely on close cooperation with local experts and would be extremely difficult to be organized by international infrastructures. A particular challenge currently being worked on in the community is the integration of national checklists with international information, such as the Catalogue of Life, with the aim of being able to link locally collected biodiversity data using taxon references (Berendsohn 2023).

### NBDIs as a data hub for national citizen science activities

Citizen science or participatory science activities form the backbone of biodiversity information on distribution of species, because, for instance, up to 90% of all biodiversity records in Europe are estimated to have been collected by volunteer efforts (Henle et al. 2013). Citizen science data are often collected either through community-based research projects with a goal of cocreating biodiversity data, information, and scientific knowledge or, largely, by natural history societies and networks linking heterogeneous structures such as nongovernmental organizations, public policy actors, individuals, and researchers with a goal of collecting biodiversity data. These volunteer data can be invaluable for the creation of red lists of threatened species or for conservation planning (Chowdhury et al. 2023). In the present article, NBDIs play an important role, particularly in supporting citizen science by providing dedicated easy-to-use tools, services, and guidelines, notably on data management including metadata (e.g., Kelling et al. 2019), as well as provision of secure data infrastructure environments. In addition, they can help to link to applications using novel technologies (van Klink et al. 2022), including AI (e.g., automated image classification as in iNaturalist). Importantly, they can also enhance the visibility of citizen science activities and organizations in national networks. The use of nationally coordinated standards and tools enables the harmonization of data from both structured and unstructured recordings, their aggregation and curation in NBDIs, and the connection to international networks for biodiversity data.

### NBDIs as mediators for legal questions

NBDIs typically do not collect any person-related data. Any such regulations, if they apply, must be followed by the data such as the data collecting scientist or a journal publishing biodiversity data in a paper. A dedicated ethics policy is currently not required for NBDIs. However, sensitive information exists in the case of geographic occurrence data for threatened species. Open access to this type of information may be used for poaching, illegal collection, and trade and could therefore possibly further harm endangered species (Lindenmayer and Scheele 2017). Database solutions exist in NBDIs allowing a consistent spatial coarsening for selected data sets (Schulmann et al. 2021). Licensing of regional data is another issue because some regional data providers follow a restrictive policy that prevents publication of these data via international data platforms (Novotný et al. 2022). On the other hand, this information is particularly important for protective policies, so many data providers provide this data openly.

National and international regulations have become particularly relevant with respect to access and benefit sharing agreements for the use of biodiversity. Since 1992, the CBD has recognized the sovereignty of individual states over their biological resources. Subsequently, in 2010, the Nagoya Protocol on Access to Genetic Resources and the Fair and Equitable Sharing of Benefits Arising from their Use to the CBD (in short, the Nagoya Protocol) defined legally binding conditions for the use of genetic resources (i.e., biodiversity) and the mechanisms for compliance, such as obtaining official permits for accessing genetic resources (Overmann and Scholz 2017). As basic research is considered a form of use, it has to comply with the regulations of the Nagoya Protocol, and compliance needs to be documented in research infrastructures, together with the data resulting from the research. To date, the Nagoya Protocol applies to physical genetic resources that include entire organisms and nucleic acids, as well as extracts containing chemical constituents, but it is, so far, not generally applied to digital data.

As nucleic acid sequence information gains increasing importance for biodiscovery and bioprospecting, many biodiverse nations perceive the free and open access to digital sequence data as a loophole that potentially prevents a fair and equitable benefit sharing from their use. As a result, several countries have requested to extend the regulations of the Nagoya Protocol to biodiversity data (so-called digital sequence information). Notably, some countries already regulate the use of biodiversity data and either do not permit uploading in public databases or impose restrictions even when biodiversity data are made publicly available through international databases such as the INSDC (the latter is the case of Brazil). Obviously, a general requirement for obtaining individual permission by the end user of biodiversity databases and the individual country of origin would ultimately prevent all larger, particularly the integrative analyses of biodiversity data.

A multilateral benefit-sharing model that maintains open access to biodiversity data while efficiently generating funds to support capacity building and the research infrastructure development in low- and middle-income countries has recently been proposed as a much more adequate solution (Scholz et al. 2022). NBDIs have specific expertise to support the current efforts to specify the multilateral mechanism of benefit sharing and solve pending issues and in fact should actively participate in this process in the years to come. They already provide major nonmonetary benefits for their partners in the Global South, through free services, training opportunities and knowledge transfer, and by handling compliance issues in the curation process. In this regard, NBDI are mediators, as well as important multipliers for legal conformity in biodiversity data management.

### NBDIs as platforms for quality control, data integration and analysis

NBDIs provide the means for a coordinated quality control, generating a broad and comprehensive data offer. Data quality and data enhancement functions can be integrated into local data curation platforms and data pipelines in a partly automated way. These pipelines not only implement processes for detecting and correcting erroneous data, but they also serve to apply controlled vocabularies and link to semantic resources that support the overarching integration of data. The platforms used must ensure that annotations are reported back to the primary data sources and that appropriate credit is given for contributions to data quality. However, manual data curation is still considered the gold standard for establishing and maintaining reliable data sets for subsequent analysis. The manual curation of taxonomic or phenotypic information relies on taxonomic and geographic expertise that is mostly found at specialist collections and natural history museums, which are typically also key partners of NBDIs—for instance, in the German NFDI4Biodiversity and GFBio (Grobe et al. 2019). The French Biodiversity Data Hub (Pôle National de Données de Biodiversité, PNDB) reuses FAIR quality assessment reports provided by DataONE network, which not only provides information on the FAIRness of existing shared data but also guides contributors to enhance the FAIRness of their own data.

*species*Link indices each data record with the status of its scientific name as *valid, synonym*, or *not found* using the GBIF taxonomic backbone and national lists as reference. Curators and specialists responsible for the data can easily identify records to be corrected or updated, and users can filter records through the search interface using these parameters. *species*Link also offers an annotation tool, where users may add annotations to a specific record, such as identifying specimens and correcting scientific names. The annotation is associated with the specific record, available to all users.

Land use and coverage is also indexed to each record collected in Brazil, thanks to the data provided by mapBiomas (https://brasil.mapbiomas.org/). All geographic coordinates informed by the collections are indexed with this information as of 1985, enabling users to produce lists of species potentially affected by land-use changes.

Furthermore, NBDIs not only facilitate data mobilization but enable novel ways of integrating biodiversity information and knowledge through their multidisciplinary nature. A multidimensional analysis of the available molecular, phenotypic, and ecological data has the potential to deduce so far unknown properties of biological species. By transforming the quality-controlled data into machine readable format, knowledge graphs can be established that allow innovative search options for the discovery of hidden data relationships. For example, AI approaches, particularly machine-learning and deep-learning technologies, can be used to extract phenological data from specimen images (Pearson et al. 2020, Weaver and Smith 2023) and can help to make predictions of phenotypic traits on the basis of molecular information (Danilevicz et al. 2022).

Finally, NBDIs have the potential to develop an integrated, highly accessible service offering from the aforementioned data quality and data integration workflows. In the case of the German NFDI, a capable, multicloud platform (the Research Data Commons) has been established as a central new component where data can be aggregated, semantically linked and enriched with external services (Glöckner et al. 2020). Support for the visualization of research data, which was addressed with the development of the Geo Engine service, plays an important role in the RDC infrastructure (Beilschmidt et al. 2023).

### NBDIs as an instrument for lobbying and structuring national funding

Next to the long-term funding by member states, international infrastructures such as GBIF, ELIXIR, and other European research infrastructures depend on the contributions of their national nodes and member organizations. On a country level, such contributions were usually stimulated through individual project grants to institutions, to cover additional costs for the participation in working groups, the connection of services to the international level, or the local rollout of agreed tools and workflows. As continuous roles for national nodes and members emerge, long-term funding needs to be secured for these as well. As organizations, NBDIs can help to articulate these needs and benefits, including them in the business cases for national funding schemes.

In Germany, many long-term projects financing national nodes were exhausted between 2016 and 2020, including the German GBIF nodes. Funding related to the NFDI, specifically the NFDI4Biodiversity project, currently helps to continue part of the activities—for example, by assisting data providers to publish their resources through GBIF. NFDI4Biodiversity also sustains user support activities in two of the method-oriented de.NBI service centers, which were previously financed as part of the German ELIXIR node, next to building working relations with the ELIXIR Biodiversity Community EuropaBON and the Barcode of Life network. As the NFDI grows and becomes more mature, it could in future serve as a hub for the upkeep of various domain-specific national nodes in international scientific data infrastructures. This was also recommended by the German Council for Scientific Information Infrastructure during the initialization of the NFDI (RfII 2017).

In the Netherlands, the GBIF node NLBIF (Netherlands Biodiversity Information Facility) is a division of the Naturalis Biodiversity Centre, following a merger in 2021. NLBIF has a program budget issuing annual calls and is coordinated by a central office team.

Through the merger with Naturalis, the Dutch GBIF node is part of the Large-Scale Research Infrastructure Roadmap of the Netherlands (NWO 2021), although it is not specifically mentioned there. Within the Large-Scale Research Infrastructure Roadmap program, the Dutch government grants funding to several research clusters every 2 years. In 2021, ARISE became part of the roadmap and received a 10-year grant (NWO 2020).

In France, the national research infrastructure roadmap is directly linked to the European Strategy Forum on Research Infrastructures (ESFRI) roadmap and fundings are oriented to support projects inline with international activities on the European Open Science Cloud (EOSC), the global Research Data Alliance, or the GO FAIR initiative. The French PNDB biodiversity infrastructure was a big player of this strategy, leading the GO FAIR BiodiFAIRse implementation network, devoted to biodiversity communities, and working directly on three EOSC-oriented European projects (EOSC-Pillar, FAIR-EASE, and EUROSCIENCEGATEWAY).

In the United States, a national committee for biological collections has presented a comprehensive report describing the importance and potential of biological collections and defining a concrete plan for their sustainable development as a research infrastructure. The report provides research funders with a framework for the financial support of biological collection data infrastructures, as well as guidance on thematic focus and prioritization (NASEM 2020).

This shows that for developing, maintaining and enhancing these links it is important to have NBDIs at a national level and to benefit from associated funding opportunities.

## Linking regional, national, and international data service levels

A key function of national data infrastructures is to connect local applications with international services. Local use scenarios are diverse and range from individual research projects and citizen science initiatives to national agencies with various reporting obligations. National data infrastructures can mediate between the specific requirements of different local and national users and the often hard to oversee services offered by international data infrastructures by providing assessments of relevant services for individual use cases, offering training in local languages and preparing and documenting data that play a special role for use in local and national applications.

Conversely, national data infrastructures can support the provision of local and national data offerings for use in an international context. In the context of NFDI4Biodiversity, for example, a service API was developed for the Red List data infrastructure coordinated by the German Federal Agency for Nature Conservation, which makes the data available for service-based applications. In a next step, local checklists will be linked to international taxonomic backbone services (e.g., EU-Nomen, Catalogue of Life) with taxonomic names and their associated identifiers as common denominators of biodiversity data, such as observations, sequences, specimens, and literature and therefore create the possibility to link national data at an international level. In Europe, the NBDI can profit from ongoing consolidation efforts regarding research infrastructure investments, specifically the ESFRI and the EOSC program.

Although national data infrastructures are by definition primarily active in their respective countries, they form the basis for enhanced international cooperation. The collaboration potential is immense. For example, existing software for national data platforms should be used and developed jointly, as is the case for the Atlas Living Australia family of NBDIs or the more specific citizen science system Les Herbonautes (http://lesherbonautes.mnhn.fr/), which is used in several countries in respective languages and adapted for different museums and taxa. Furthermore, it can also be useful to cooperate on consultancy services—for example, by exchanging or jointly developing materials.

Joint discussion platforms, such as the symposium National Biodiversity Data Centers: Challenges and Opportunities during the TDWG2022 conference are helping to formulate common goals and coordinate the implementation of measures. Accompanying joint working groups will help to plan and implement projects at the organizational and technical level.

For effective interaction between local, national, and international levels, a clear division of tasks and definition of the respective roles is essential. National data infrastructures are crucial to serve as facilitators, ambassadors, mediators, and platforms to provide the identified 10 essential functions. They are ideally positioned to link the relevant actors at all levels and to define the respective tasks and the required interfaces.

## Outlook

Many NBDIs are continuously adapting to the growing needs of standardization, scaling, and adding functionality supporting biodiversity related data. In many cases, although their original focus was around the national natural history collections, continuous development is needed. This especially concerns new technologies, such as environmental DNA, sensors including Internet of things devices and remote sensing via drones and satellites, imaging technology, and AI. As a consequence, recent interest has grown rapidly, as can be seen from the multitude of funded projects and initiatives in this area—for example, on a global level, GEOBON (Scholes et al. 2012) and the International Barcode of Life and, on a European level, MAMBO (Modern Approaches to the Monitoring of Biodiversity), InsectAI, Biodiversa+. Although many of these technologies are now implemented at the research institute level they are also becoming available at the national level. ARISE is one of the frontrunners as NBDI for the Netherlands (van Ommen Kloeke et al.^ 2022^). It has already established end-to-end capabilities for species recognition on the basis of DNA and AI for image and sound recognition and is expanding its capacity to enable technology based monitoring at scale. It has the ambition to facilitate a platform to build, improve, share, and run any AI model and connect any sensor. Adopting such technology also opens up collaboration across domains, with especially the computer science domain participating as direct collaborators.

With the work presented in the present article, national data infrastructures have jointly begun to define tasks and objectives that should be addressed at national level. However, the national focus does not mean that these tasks are being worked on in isolation; rather, greater cooperation is desirable and contributes to the effective implementation of objectives. At the organizational level, the establishment of an interest group (e.g., under the umbrella of TDWG or the Research Data Alliance) could be used to develop a common agenda and integrate further national initiatives. Concrete measures to strengthen the 10 functions defined in the present article, can then be coordinated within the framework of this organizational structure. These range from the creation of joint forums and documentation platforms—for example, on legal aspects, data quality methods, including those evaluating and adopting the latest technologies such as AI, to the joint development and operation of resources and services required at national level.

## Conclusions

In recent years, more and more NBDIs have been established worldwide that serve as much needed national hubs for national biodiversity knowledge and provide professional tools and link to international data services. Experience shows that the respective orientations of NBDIs can be very different and range from very specific infrastructure measures to the creation of comprehensive data platforms that strengthen national research data management as a whole. Despite the different approaches, all national data infrastructures close the gaps between local actors (academia, citizen science, museums, etc.) and the globally operating data infrastructures. The 10 most important roles for NBDIs include to serve as providers of base infrastructure, facilitators for data mobilization, facilitators for concerted national digitization efforts, ambassadors for the application of standards, local helpdesks for using international data infrastructures, facilitators for linking regional biodiversity assessments and taxonomies, data hub for national citizen science activities, mediators for legal questions, platforms for quality control, data integration and analysis, and an instrument for lobbying and structuring national funding.

NBDIs form essential national nodes for science, policy, and practice needs by enabling and enhancing joint data mobilization and automated curation, data storage, and archiving to international standards that allows modern analyses of interoperable data for both pure and applied science, as well as for biodiversity policy and management. By identifying the 10 essential functions of NBDIs we hope to guide and foster new and ongoing developments of national structures to strengthen and secure high profile biodiversity science, as well as evidence-based policy and management to reach the GBF targets.

## Author Biography

Anton Güntsch (a.guentsch@bo.berlin) is the director of the Center for Biodiversity Informatics and Collection Data Integration at Botanic Garden and Botanical Museum Berlin, Freie Universität Berlin in Berlin, Germany. Jörg Overmann (joerg.overmann@dsmz.de) is the scientific director of the Leibniz Institute DSMZ-German Collection of Microorganisms and Cell Cultures and also holds a chair of Microbiology at the Technical University of Braunschweig. Barbara Ebert (barbara.ebert@gfbio.org) is the executive secretary of the German Federation for Biological Data and the NFDI4Biodiversity consortium. Aletta Bonn (aletta.bonn@idiv.de) is a professor and head of department/research group Biodiversity and People at the Helmholtz-Center for Environmental Research - UFZ, Friedrich Schiller University Jena and the German Center for integrative Biodiversity Research (iDiv). Yvan Le Bras (yvan.le-bras@mnhn.fr) is the scientific and technical coordinator of the French Biodiversity Data Hub (PNDB) e-infrastructure, and located at PatriNat support and research unit, French Museum of Natural History, Concarneau marine station, France. Dora Anne Lange Canhos (dora@cria.org.br) is the associate director of CRIA - Centro de Referência em Informação Ambiental and part of speciesLink's technical team in Campinas, São Paulo, Brazil. Thore Engel (thore.engel@idiv.de) is a researcher in the research group Biodiversity and People at the Friedrich Schiller University Jena, the German Center for integrative Biodiversity Research (iDiv) and the Helmholtz-Center for Environmental Research - UFZ. Knut Anders Hovstad (knut.hovstad@artsdatabanken.no) is developing systems for management and communication of species occurrence data at the Norwegian Biodiversity Information Centre, and an affiliated member of the Centre for Biodiversity Dynamics, Norwegian University of Science and Technology, Norway. Peggy Newman (peggydnewman@gmail.com) is the data manager of the Atlas of Living Australia, based at the Commonwealth Scientific and Industrial Research Organization (CSIRO) in Melbourne. Elaine van Ommen Kloeke (elaine.vanommenkloeke@naturalis.nl) is the program manager of ARISE, located at the Naturalis Biodiversity Center in Leiden, the Netherlands. Sophia Ratcliffe (sophia.ratcliffe@posteo.net) worked for the NBN Trust as the NBN Atlas Data Manager. Marianne le Roux (m.leroux@sanbi.org.za) is the e-Flora coordinator of South Africa project at the South African National Biodiversity Institute, in Pretoria, and a research associate at the University of Johannesburg, in Johannesburg, South Africa. Vincent S. Smith (v.smith@nhm.ac.uk) is head of Digitial, Data, and Informatics at the Natural History Museum, in London, England, in the United Kingdom. Dagmar Triebel (triebel@snsb.de) is affiliated with the State Collection for Botany and the SNSB IT Center Core Facility at the Bavarian Natural History Collections, Germany. David Fichtmueller (d.fichtmueller@bo.berlin) is a researcher at the Center for Biodiversity Informatics and Collection Data Integration at Botanic Garden and Botanical Museum Berlin, Freie Universität Berlin in Berlin, Germany. Katja Luther (k.luther@bo.berlin) is a researcher at the Center for Biodiversity Informatics and Collection Data Integration at Botanic Garden and Botanical Museum Berlin, Freie Universität Berlin in Berlin, Germany.
